# Emergence of a febrile illness of unknown causes among the population and visitors of Upper Egypt

**DOI:** 10.11604/pamj.supp.2021.40.2.30988

**Published:** 2021-12-16

**Authors:** Maged Mounir Ibrahim, Afreenish Hassan Amir, Ricardo Strauss, Muhammad Asif Syed, Eva Mertens

**Affiliations:** 1 Central Laboratory for Evaluation of Veterinary Biologics (CLEVB), Agriculture Research Center (ARC), Cairo, Egypt,; 2 National Institutes of Health, Islamabad, Pakistan,; 3 Bernhard Nocht Institute for Tropical Medicine, Department of Infectious Disease Epidemiology, Bernhard-Nocht-Strasse 74, 20359 Hamburg, Germany,; 4 Field Epidemiology Laboratory Training Program (FELTP) Pakistan, Islamabad, Pakistan,; 5 Global Partnership Initiated Biosecurity Academia for Controlling Health Threats (GIBACHT), Hamburg, Germany

**Keywords:** Case study, biosafety, biosecurity, outbreak investigation, epidemiology, surveillance

## Abstract

This is a fictional case study for training that encourages participants to interact and apply theory into practice. A febrile illness of unknown cause that occurred in Upper Egypt in 2002 was chosen for the events. The location of Aswan was selected to define the climate, topography, and location with the characteristics that support the events. Data obtained from applied research work in Egypt was included. The case study deals with the incidence of severe cases of fever of unknown origin accompanied by neurological and intestinal symptoms, as well as a high percentage of deaths. Most of the symptoms appear in people with direct contact with farm animals especially equines and birds, or those who were near waterways, either tourism workers or tourists. Most of the infected cases or deaths have accumulated in Aswan and some in the neighbouring governorates. This case study focusses on the steps taken during an outbreak investigation, and deals with investigative challenges as well as concepts of biosafety and biosecurity.

## How to use this case study

### General instructions

This is a fictional case study and some of its real data was obtained from an applied research work and investigations in Egypt [1]. The details of the original research work have been modified to enhance learning objectives and support the instructional goal. Fifteen (15) participants should be matched to two facilitators in a training room, preferably at a round table. Each participant should be issued a copy of the case study prior to its implementation. The facilitator guide remains with the facilitator. The facilitator introduces the learning objectives of the case study and materials that participants should have at hand before starting the case study. Participants take turns to read the case scenario and the questions or tasks that follow. The participants then discuss and attempt to provide the answer to the question. The facilitator should stimulate participants to discuss and arrive at the correct responses.

### Audience

Health care providers, epidemiologists, clinicians, undergraduate/ postgraduate students and public health professionals.

### Prerequisites

Before using this case study, participants should have received lectures or other instruction in outbreak investigation.

### Materials

Note book, pen, flipcharts, chart papers, markers, calculators and laptops.

### Level of training and associated public health activity

Novice - Outbreak investigation

### Time required

The expected time for the case study is about 3 hours

### Language

English

## Case study material

- Download the case study student guide (PDF - 1.38 MB)
- Request the case study facilitator guide: contact info@gibacht.org
